# Malaria Tropica: An Autopsy Case Report With a Discussion on the Presence of Malaria in Bulgaria

**DOI:** 10.7759/cureus.61862

**Published:** 2024-06-06

**Authors:** George S Stoyanov, Lilyana Petkova, Hristo Popov

**Affiliations:** 1 Pathology, Multiprofile Hospital for Active Treatment, Shumen, BGR; 2 Pathology, Complex Oncology Center, Shumen, BGR; 3 General and Clinical Pathology, Forensic Medicine and Deontology, Medical University of Varna, Varna, BGR

**Keywords:** plasmodium falciparum, malignant malaria, blackwater fever, bulgaria, histopathology, autopsy, malaria

## Abstract

Malaria is an infectious disease caused by several types of parasitic plasmodia and transmitted to humans through Anopheles mosquitoes. The disease has long been widespread and has caused a significant number of deaths and decreased life quality from sequelae worldwide. As understanding of the disease increased immensely at the beginning of the 20th century, eradication plans were implemented to decrease disease transmission. This led to the successful eradication of malaria across predominantly industrialized countries, with multiple geographic areas remaining malaria endemic zones to this day. With climate changes and migration, the risk of reintroduction of malaria to malaria-free zones has risen due to relatively easy travel to endemic zones and importation of cases. On the one hand, this is a significant public health risk and, on the other, a challenge to the medical system, as healthcare workers in malaria-free zones are often ill-prepared to recognize, diagnose, and treat malaria cases. Herein, we present an autopsy and histopathology case report of tropical (falciparum) malaria, complicated with blackwater fever (malignant malaria) with prevalent gross and histopathological changes, including hemomelanin deposition in the spline, liver, and bone marrow; visible parasitic forms in the remaining red blood cells; Durk's granulomas, sludge, and petechial hemorrhages in the central nervous system; and hemoglobin casts within the renal tubular structures. We also discuss the history and risk of reintroducing malaria into a malaria-free zone - Bulgaria.

## Introduction

Malaria is an infectious disease caused by parasitic microorganisms from the Plasmodium group [1,2]. Currently, five members of plasmodia are known to cause disease in humans - falciparum, vivax, ovale, malariae and knowlesi - with each differing in their clinical presentation, form, severity, and rate of complications [2,3]. Among these, Plasmodium falciparum causes the most severe form of malaria, classically referred to as malaria tropica, with vivax and ovale causing tertian malaria (tertian fever), which is less severe, and Plasmodium malariae causes quartan fever [4]. Plasmodium knowlesi is the least likely of the plasmodia to cause disease in humans and causes the least severe clinical form of all [5].

Infection typically occurs via a vector mechanism, with mosquitos from the Anopheles genus acting as vectors [3,6,7]. Other forms, such as vertical and transfusion-associated malaria, also occur but are extraordinarily rare [8-10].

Malaria has accompanied humanity for most of its evolutionary history but became widespread and recognized as a significant health risk during the latter part of the Middle Ages and especially during and after the Industrial Revolution [4]. Death due to infection and decreased life quality due to recurrent non-lethal infections lead to many efforts to lessen the impact of malaria, both in its treatment and the eradication of its vector and the conditions in which it reproduces [1,11,12]. These efforts were widely successful in industrially developed countries; however, multiple regions remain endemic zones for malaria, and with the decrease in efforts to control malaria, its vector has been reintroduced into malaria-free zones [13,14]. With the availability and widespread of travel to endemic zones and migration from them, there is a high risk for the reintroduction of malaria into malaria-free zones, which will inevitably lead to severe consequences due to medical staff in these areas being generally inexperienced in recognizing, diagnosing, and treating malaria [13,14].

The clinical course of malaria, dependent on the Plasmodium, is based on the reproduction of the parasites in red blood cells, which rupture upon maturation of newly produced parasites and lead to infection of new ones [2,3]. The lysis of red blood cells leads both to anemia and acute toxicosis, based on the release of toxic metabolites and infection of new red blood cells as well as damage to multiple systems - liver, spleen, and bone marrow, with an accumulation of byproducts in macrophages; kidneys - with acute tubular dystrophy leading acute kidney failure; and central nervous system - with vascular and parenchymal damage [1,5,11,12,15].

Herein, we present an autopsy case report of tropical malaria from a malaria-free zone and discuss the history and possibility of reintroduction of malaria in Bulgaria.

## Case presentation

A previously healthy 36-year-old male patient presented to our institution with new-onset fever and general malaise for the past week. Imaging modalities did not show identifiable foci of inflammation, with laboratory tests showing signs of hemolysis and elevated liver enzymes. History was significant for recent travel to central Africa (two weeks prior), due to which parasitological workup for malaria was initiated. Parasitology confirmed Plasmodium falciparum in blood smears and treatment for malaria tropica was initiated, on the background of which the patient progressed with severe hemolysis to the classical presentation of blackwater fever; jaundice and drowsiness also developed, on the background of which the patient progressed to classic malaria coma and expired 16 after the initiation of treatment.

Prior to the autopsy, on observation, mild obesity was noted; however, body mass index was not measured, and no further external signs were noted apart from jaundice and pale livor mortis. In the section of the thoracic cavity, the pleural sheets were edematous with scattered petechial hemorrhages. Probes for gas/air embolism were negative. The lungs were hyperemic (left lung of 900 g, right lung of 1,000 g), with a rusty red to gray-blue color in some areas. The mucous membranes of the trachea and bronchial three were preserved, with the pulmonary cut surface being variegated, and when compressed, a copious amount of frothy pink liquid flowed spontaneously. The vascular and bronchial trees were free from obstruction. The sheets of the pericardial sac were smooth with scattered petechial hemorrhages. The heart had a rounded apex (weight of 680 g and thickness of the left ventricular wall of 25 mm and of the right one of 5 mm), and the valvular cusps had normal morphology. Cardiac chambers had a marked topography of the trabecular musculature, and the myocardium had a preserved bundled structure in cross-sections.

**Figure 1:**
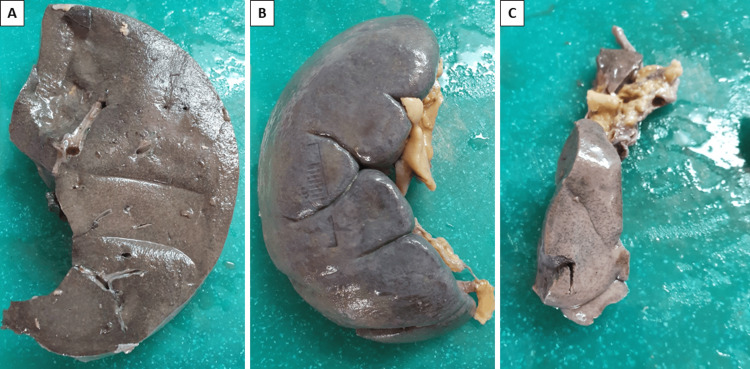
Gross view of the sections of the liver (A), spleen (B), and kidney (C) post fixation in formaldehyde

Sectioning of the digestive system structures revealed a preserved mucosa of the esophagus, stomach, and intestines. The liver was significantly enlarged (2700 g), pale grey to yellow, with a homogeneous cut surface (Figure 1A). The gallbladder was free of stones, and the bile ducts were free from obstruction and stenosis. The pancreas had a finely lobulated, mottled surface.

The spleen was significantly enlarged (630 g), with a preserved shape and localization, gray-violet in color (Figure 1B). The cut surface had the same gray-violet appearance, was dense, and did not scrape off with the back of the knife; several wedge-shaped pale and loose areas were noted on the lower pole.

The two kidneys were relatively the same size (left one of 270 g, right one of 250 g) and easy to decapsulate, cyanotic to pale grey (Figure 1C). Their cut surface was smooth, with a preserved cortico-medullary differentiation. The pelvises and ureters had a preserved mucosal lining and were free from obstruction. The bladder was filled with a moderate amount of dark-yellow urine and lined with normal mucosa.

Endocrine glands appeared grossly normal; genitalia were male and adequately developed for the age.

**Figure 2:**
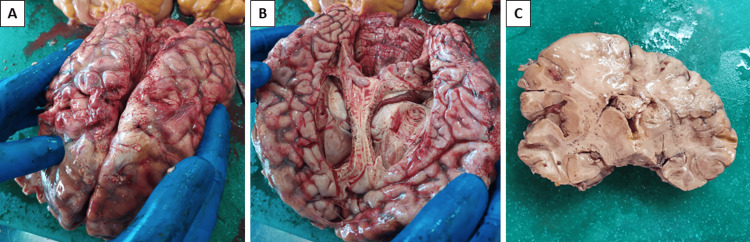
Gross view of the cerebrum and meninges (A), petechial hemorrhages on the section (B) in the fresh specimen, and (C) after fixation in formaldehyde

Sectioning of the cranium revealed edematous meninges. Cerebral hemispheres were symmetrical and edematous (brain weight of 145 0g) (Figure 2A). On section, the cut surface was variegated, and at the level of each cut, diffusely in the white matter, numerous petechial hemorrhages were seen, which did not scrape off with the back of the knife (Figures 2B-2C). The ventricles were symmetrical, filled with clear cerebrospinal fluid, and lined by edematous ependyma. The cerebellar tonsils showed well-formed furrows of symmetrical wedging in the large occipital foramen.

Acute fractures were noted in the second to fifth ribs on the left as a result of cardiopulmonary resuscitation (CRP).

Histologically, the lungs showed acute venous stasis, focal acute hemorrhages, and intraalveolar edema. Intravascularly, intraerythrocytic ring forms of plasmodia (schizonts) were visible within the erythrocytes, as well as a brown granulated pigment - hemomelanin (hemozoin), which was abundant in erythrocytes, extracellularly and in macrophages. Some foci of the lung, predominantly peripheral ones, showed hyaline microcirculatory thrombi, and a single vessel had evidence of bone marrow embolism, which further supported the finding of CRP-associated fracturing of the ribs. The grossly firm and reddish lung areas showed focal signs of pulmonary proteinosis - hyaline membranes on histopathology.

**Figure 3:**
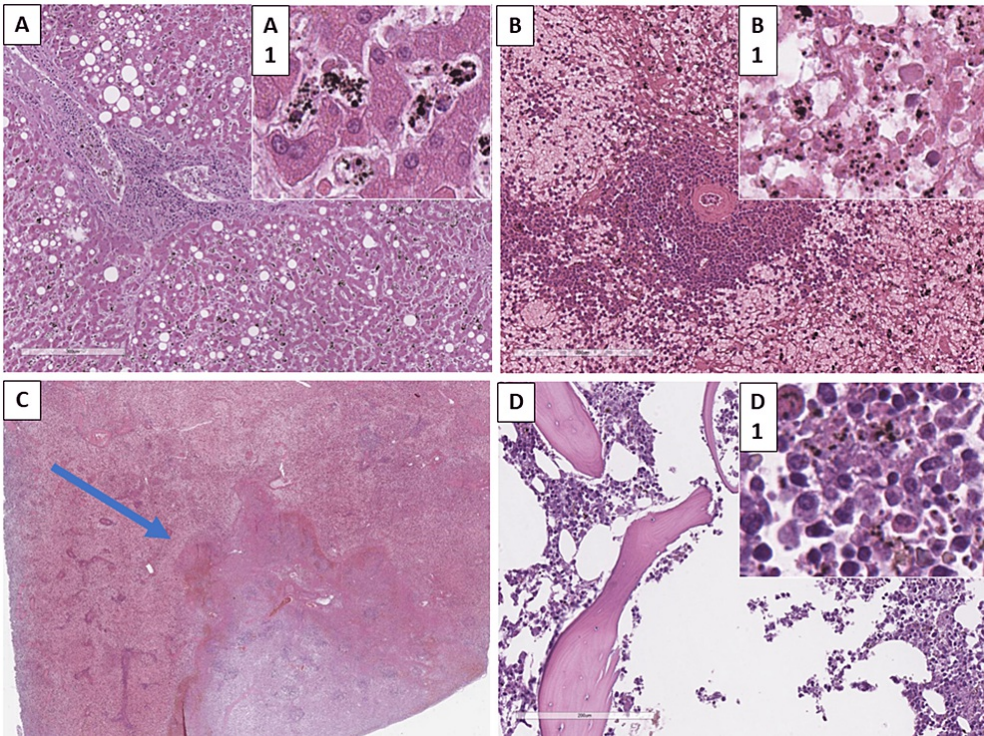
Histopathological changes in the liver, spleen, and bone marrow A: liver with foci of portal inflammation, steatosis, and foci of pigment deposition, H&E stain, original magnification 100x, A1 original magnification 400x; B: spleen with stasis and foci of pigment deposition, H&E stain, original magnification x200, B1 original magnification 400x; C: spleen with foci of infarction (arrow), H&E stain original magnification x10; D: bone marrow with proliferation of the hematopoietic parenchyma and foci of pigment deposition, H&E stain, original magnification x200, D1 original magnification 400x. H&E: hematoxylin and eosin

The liver showed signs of intra- and extracellular cholestasis; hemomelanin was also present both extracellularly and in macrophages, which, under polarizing, gave a green birefringence, unlike the bile pigments that were also present (Figure 3A). Hyperplasia of Kupffer cells was also noted, most of which were laden with hemomelanin; chronic portal inflammation, micro-macrovesicular hepatosteatosis, and acute venous stasis with visible trophozoites in the erythrocytes were also noted. The rest of the gastrointestinal structures did not show any pathological changes.

The spleen showed hyperplasia of the red pulp, acute infarctions with a prevalent hyperemic-hemorrhagic margin, acute venous stasis with focal acute hemorrhages, plasmodia (schizonts) in erythrocytes, and free and absorbed by macrophages' hemomelanin (Figures 3B-3C). The bone marrow had a preserved trabecular structure with hypercellular hematopoietic parenchyma due to erythroid lineage hyperplasia and an abundance of hemomelanin-laden macrophages (Figure 3D).

**Figure 4:**
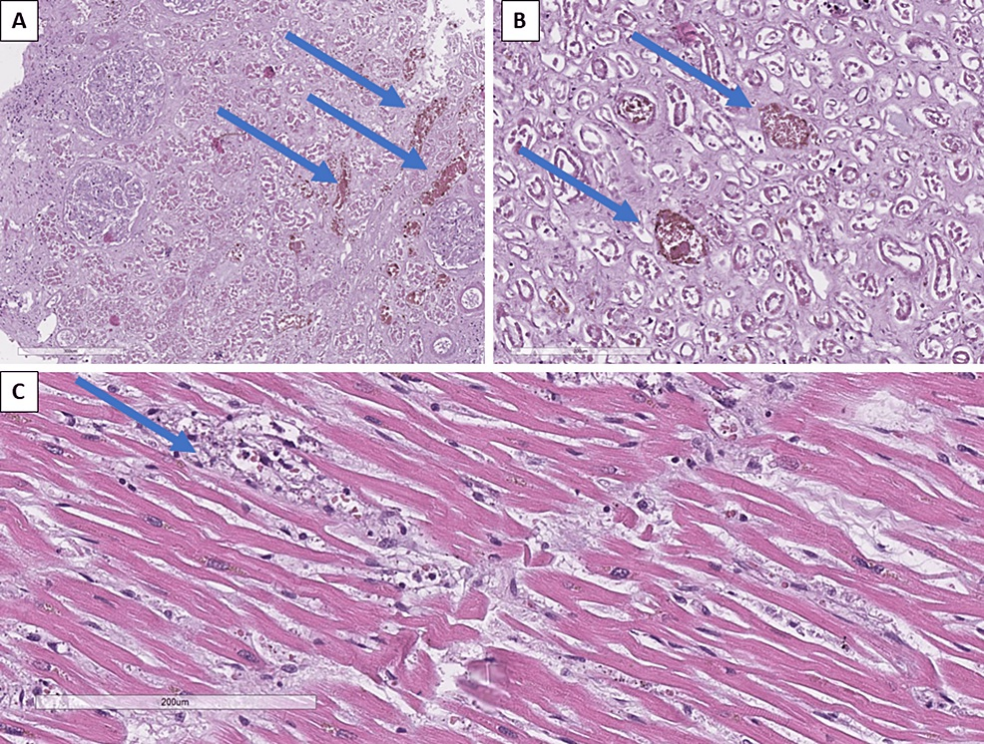
Histopathology changes in the kidneys and heart A: hemoglobin casts in the tubular system of the kidneys (arrows), H&E stain original magnification x100; B: tubular degeneration and hemoglobin casts (arrows) in the tubular apparatus of the kidney, H&E stain, original magnification x200; C: interstitial inflammation (arrow) and fibrosis within the myocardium, H&E stain, original magnification x200 H&E: hematoxylin and eosin

The kidneys showed tubulo-epithelial degeneration, hemoglobin casts in the tubular apparatus, and acute venous stasis (Figures 4A-4B). The ureters and urinary bladder had a preserved histological structure.

The myocardium showed cardiomyocyte hypertrophy, focal cardiomyocyte degeneration with an inflammatory reaction in the interstitium, predominantly lymphocytic infiltrates and focal hemorrhages in the subepicardial adiposa, mild interstitial and perivascular fibrosis, as well as acute venous stasis with malarial plasmodia visible in the erythrocytes (Figure 4C).

The endocrine structures had a predominantly preserved morphology, apart from focal microhemorrhages in the cortex of the adrenal gland and parenchyma of the anterior pituitary gland.

**Figure 5:**
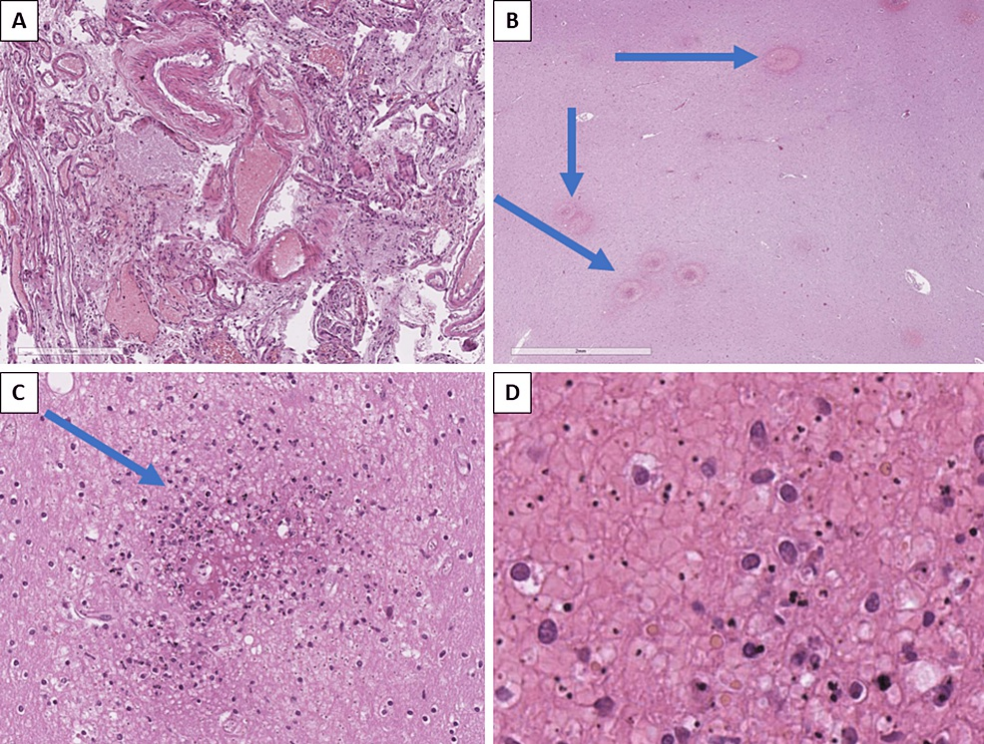
Histopathological changes in the central nervous system structures A: leptomeningeal edema and stasis - sludge phenomenon, H&E stain, original magnification x100; B: petechial hemorrhages (arrows), H&E stain, original magnification x100; C: Durk granuloma (arrow), H&E stain, original magnification x200; D: plasmodia in congested erythrocytes, H&E stain, original magnification x400 H&E: hematoxylin and eosin

The central nervous system showed arteriolohyalinosis and sclerosis, pronounced pericellular, perivasal and leptomeningeal edema (Figure 5A), acute venous stasis, petechial hemorrhages, small-focal perivascular leukomalacia foci (Figure 5B), Durk granulomas (foci of microglial proliferation and aggregation) (Figure 5C), plasmodia in erythrocytes in vessels, hemomelanin in erythrocytes (Figure 5D), and neuronal degeneration.

Based on the broad spectrum of observed malaria-associated gross and histopathology changes and the parasitological proof of P. falciparum, the cause of death was concluded as complicated tropical malaria (blackwater fever).

## Discussion

**Figure 6:**
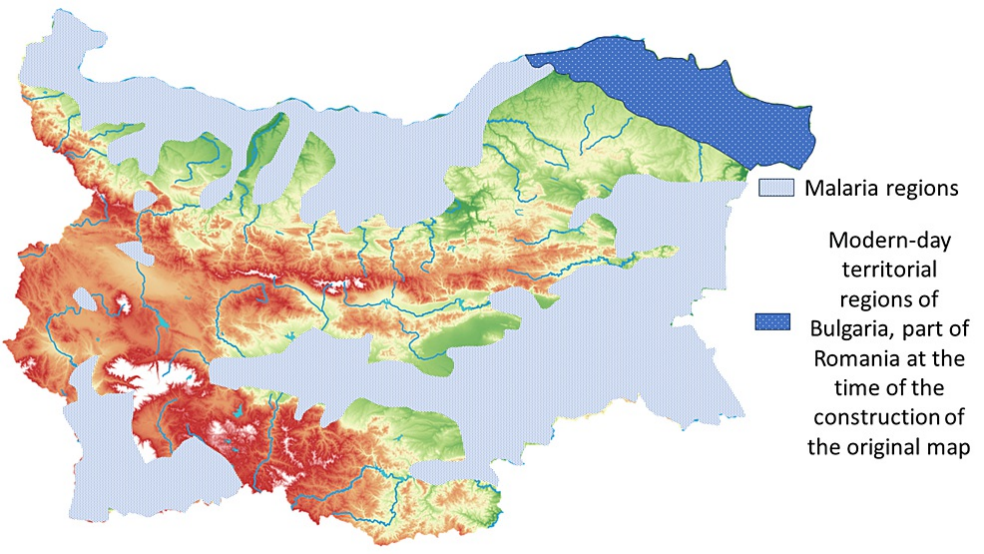
Distribution of malaria regions in Bulgaria in 1940 Adapted from [11]. Note: Due to the timeframe in which the original map was produced, the spread of malaria in the then-Romanian regions is unknown.

Malaria has long been a disease distributed across lands inhabited by Bulgarians [11]. In some regions, the spread was so widespread that these endemic areas were not even required by legislation after the liberation in 1878 to report malaria incidence [16]. Entering into the 20th century, after several regional conflicts and especially after the First World War, due to decreased efforts on control and the general decrease in the population's life quality, malaria became a seasonal and almost universal disease even in large cities (Figure 6) [11,12,15]. In one of the largest cities in the country - Varna - in 1901, from 13,237 patients that presented to ambulatory check that year, a total of 4,552 were diagnosed with malaria, or a percentage of 34.3% from all healthcare-seeking individuals, with the total city population being no more than 35,000 [16]. From 1905 to 1911, the yearly incidence of malaria in the same city varied from 1,648 to 7,952 confirmed cases, which is by far the most common infectious disease registered [1,11,12,15]. Malaria was also widespread within the military divisions, with infection rates being between 9.46% and 58.01% from all military personnel within a single division in 1916 and 1917, with malaria being the cause of death of 0%-1.12% of the total military personnel [1,11,12]. Following the First World War, a national eradication plan was implemented to reduce marshlands, chemically eradicate vector mosquitoes, and control the spread and effects of the disease with strict control of infected individuals with on-site diagnostic and treatment teams, including mobile ones for smaller cities and villages [1,11,15]. As such, the infection rates and overall mortality dropped, with the rates of tropical malaria dropping to less than 10% of all malaria cases [11]. Apart from state-run malaria institutes (a total of nine established in different malaria-affected regions), a malaria institute in Burgas was founded and funded by the Rockefeller Institute and tasked with controlling the spread of malaria in one of the most affected regions [1,11,15]. Estimates of the funding place the total subsidy amount from the Rockafeller Institute at around 100 million leva for its nearly 20-year existence, compared to only around a million leva annually for each of the remaining nine institutes (not adjusted for inflation) [11,12,15,17].

In the following decades, with some exceptions due to a complication in international affairs and the withdrawal of the Rockafeller Institute from Burgas, malaria cases gradually declined further. With the enactment of international malaria control plans and further funding, the last cases were registered as follows: Varna, Vidin, and Mihailovgrad (currently Montana) regions in 1957; Blagoevgrad, Vratsa, Kurdzhali, Plovdiv, Sara Zagora, and Veliko Turnovo regions in 1958; Kyustendil, Pernik, and Pleven regions in 1959; Burgas and Haskovo regions in 1960; and Yambol in 1961 [17]. Following several stable years without newly infected cases, the World Health Organization declared Bulgaria a malaria-free zone in 1965, despite several reports depicting chronic malaria as a cause of death in these years and the following decade. Imported cases were common, ranging from less than 10 a year in the 2000s to more than 400 in 1981 [6,7,17,18]. While initially imported cases were not considered viable for the reintroduction of malaria due to the lack of vector mosquitoes, through decreased insect management and global warming, anopheles mosquitoes have largely re-inhabited their previous areas, giving the possibility for the reintroduction of malaria in previous endemic zones from newly imported cases [7,13,14,18].

While our case depicts a classical clinical manifestation, epidemiological profile, and gross as well as histopathological features, it also underlines several peculiarities of tropical malaria. First of these is the imported nature of the case in a zone that has long been considered malaria-free by the World Health Organization, underlining that malaria-free recognizes no local infection and the current age of relatively easy travel to endemic regions is a risk factor both for individual infection and the reintroduction of malaria into such zones [13,14,18]. Secondly, our case presents a relatively rarely observed phenomenon of malaria-associated myopericarditis [19,20]. The genesis of myopericarditis in malaria is not as well-understood as its other organ-specific complications. It is also probably due to a combination of factors, including the mechanical blockage of the cardiac microcirculation, resulting in micro ischemic foci, as well as a toxic mechanism both from the malaria-associated circulating toxic byproducts and the local effect of toxic byproducts and proinflammatory cascade activation from the ischemic zones of the myocardium [19,20].

Lastly, our case underlines the severity of falciparum-associated malaria, also referred to as tropical malaria [3,11]. The most severe cases, with multiple system and organ involvement and complications, have classically been depicted as malignant malaria and blackwater fever [1,11,12,15]. The underlying mechanism in both untreated and treated cases is severe hemolysis, leading to significant hemoglobinuria and acute kidney failure [11]. As the hemoglobin content in the urine increases significantly and the iron oxidizes, the urine turns dark brown to nearly black in color, hence the term blackwater fever [11]. However, black urine fever is a more accurate description [11]. As the condition of these patients rapidly deteriorates and progresses to death due to acute kidney failure from hemoglobinuria and toxemia shortly thereafter, some authors and schools have classically depicted this condition as a malignant form of malaria. However, nowadays, the condition is reversible, and many patients survive.

## Conclusions

The presented case depicts the gross and histopathological aspects of tropical malaria caused by P. falciparum. The case, while depicting the classical organ complications of tropical malaria - liver, spleen, and bone marrow involvement by deposition of hemomelanin, cerebral vascular hyperemia with petechial hemorrhages and Druk's granulomas, hemoglobin cast in the renal tubular system, and the rarely reported myopericarditis associated with malaria - bears with it several keynotes. It also depicts the possibility of the reintroduction of malaria into zones that have long been considered malaria-free and the possible severe consequences for the healthcare systems and the health of the population as a whole, as malaria is rarely if ever, suspected, even if the clinical manifestations are classical.
